# Relative cerebral hyperperfusion during cardiopulmonary bypass is associated with risk for postoperative delirium: a cross-sectional cohort study

**DOI:** 10.1186/s12871-019-0705-y

**Published:** 2019-03-09

**Authors:** Marcus Thudium, Richard K. Ellerkmann, Ingo Heinze, Tobias Hilbert

**Affiliations:** 1 Department of Anesthesiology and Intensive Care Medicine, University Hospital Bonn, Sigmund-Freud-Strasse 25, 53127 Bonn, Germany; 2 Department of Anesthesiology and Intensive Care Medicine, Dortmund Hospital, Beurhausstrasse 40, 44137 Dortmund, Germany

**Keywords:** Cerebral blood flow, Delirium, Cardiac surgery, Cardiopulmonary bypass, Transcranial Doppler sonography

## Abstract

**Background:**

Our objective was to evaluate if changes in on-pump cerebral blood flow, relative to the pre-bypass baseline, are associated with the risk for postoperative delirium (POD) following cardiac surgery.

**Methods:**

In 47 consecutive adult patients, right middle cerebral artery blood flow velocity (MCAV) was assessed using transcranial Doppler sonography. Individual values, measured during cardiopulmonary bypass (CPB), were normalized to the pre-bypass baseline value and termed MCAV_rel_. An MCAV_rel_ > 100% was defined as cerebral hyperperfusion. Prevalence of POD was assessed using the Confusion Assessment Method for the Intensive Care Unit.

**Results:**

Overall prevalence of POD was 27%. In the subgroup without POD, 32% of patients had experienced relative cerebral hyperperfusion during CPB, compared to 67% in the subgroup with POD (*p* < 0.05). The mean averaged MCAV_rel_ was 90 (±21) % in the no-POD group vs. 112 (±32) % in the POD group (p < 0.05), and patients developing delirium experienced cerebral hyperperfusion during CPB for about 39 (±35) min, compared to 6 (±11) min in the group without POD (*p* < 0.001). In a subcohort with pre-bypass baseline MCAV (MCAV_bas_) below the median MCAV_bas_ of the whole cohort, prevalence of POD was 17% when MCAV_rel_ during CPB was kept below 100%, but increased to 53% when these patients actually experienced relative cerebral hyperperfusion.

**Conclusions:**

Our results suggest a critical role for cerebral hyperperfusion in the pathogenesis of POD following on-pump open-heart surgery, recommending a more individualized hemodynamic management, especially in the population at risk.

**Electronic supplementary material:**

The online version of this article (10.1186/s12871-019-0705-y) contains supplementary material, which is available to authorized users.

## Background

An ageing population with increasing prevalence of cardiovascular diseases results in an increased caseload of cardiac surgery as well. Postoperative delirium (POD) is one major complication following cardiac surgery. It is known to increase length of intensive care unit (ICU) and hospital stay and postoperative mortality, thus representing a significant problem for the health care system [1]. Its pathophysiology, albeit still remaining elusive, is multifactorial [2]. Although POD is usually supposed to be associated with intraoperative periods of hypotension, there is some evidence suggesting the significance of sustained cerebral hyperperfusion during cardiopulmonary bypass (CPB) [3]. In a recent work, Hori et al. demonstrated the association of an arterial blood pressure above the upper limit of cerebral autoregulation with POD, suggesting cerebral hyperperfusion to be present in these patients [4].

Superior to blood pressure monitoring, transcranial Doppler sonography (TCD) offers the possibility to directly assess blood flow velocity in the middle cerebral artery (MCA), which is an indirect measurement of cerebral blood flow (CBF) [5]. We hypothesized that changes in on-pump CBF, relative to the pre-bypass baseline value, both measured by TCD, are associated with the prevalence of postoperative delirium. Our results may underline the importance of a more individualized hemodynamic management during cardiac surgery.

## Methods

Aim was to evaluate if changes in on-pump cerebral blood flow, relative to the pre-bypass baseline, are associated with the risk for POD following cardiac surgery. This cross-sectional cohort study was conducted in accordance with the Declaration of Helsinki and after approval by the institutional review board of the University of Bonn (protocol number 300/16, date of approval 2016-06-16). According to the approval and due to the observational design of the study, informed consent was waived. Inclusion criteria were: patient age > 18 years and elective on-pump open heart surgery. The exclusion criteria were as follows: emergency procedure, aortic arch surgery with circulatory arrest, pregnancy, and absence of a proper acoustic window. All patients received anesthesia induction according to standard procedures including arterial line, intubation, central venous catheterization, and urinary catheter. Anesthesia was induced with sufentanil, etomidate, and rocuronium and was maintained with sevoflurane and continuous infusion of sufentanil. The patients received noradrenaline as vasopressor and dobutamine for inotropic support (the latter not during CPB). Median sternotomy was performed in apnea. After systemic heparinization and cannulation of the ascending aorta and of the right atrium or the superior and inferior vena cava, respectively, CPB was established using a roller pump (Advanced Perfusion System 1, Terumo Corporation, Tokyo, Japan) and a membrane oxygenator (Quadrox-I Adult, Maquet Getinge Group, Rastatt, Germany). The heart-lung machine (HLM) system was primed with 1,200 ml of crystalloid infusion solution and 10,000 IU of heparin. After full HLM support was installed with a pump flow rate of 2.5 l/m^2^ body surface area*min, ventricular fibrillation was induced, and cardiac arrest was achieved by administering Calafiore warm blood or Bretschneider cardioplegia infusion. The aorta was then cross-clamped. Mild hypothermia (32–34 °C) was induced, and acid-base management was performed according to pH-stat regimen. Towards the end of the surgical procedure, patients were rewarmed and subsequently weaned from the CPB after de-clamping of the aorta and sufficient reperfusion. After successful weaning from the CPB, heparin was antagonized with protamine in an 80–100% dose of the initially administered heparin. All patients were transferred to ICU for postoperative care.

All patients received monitoring of bispectral index (BIS; BIS vista, Medtronic Minimally Invasive Therapies, Minneapolis, USA). Baseline TCD measurement of the right MCA was performed after anesthesia induction and before establishing CPB. A S5–1 transducer on a CX30 ultrasound system (Philips Medical Systems, Hamburg, Germany) was used. Measurements were performed via a temporal window, as it was previously described by Aaslid et al. (see Additional file 1: Figure S1, Supplemental Digital Content) [5]. First, the intracranial cavity was visualized in B-mode with a depth of 15 cm, then depth was reduced to about 8 cm. Color Doppler imaging was added, and the right MCA and the Circle of Willis were visualized. Once an appropriate angle was achieved, pulsed-wave Doppler was added on the M1 segment of the MCA in a depth between 4.5 and 5.5 cm, and averaged blood flow velocity in MCA (MCAV) was measured. After aortic cross-clamping, MCAV was monitored every 10 min during CPB, and these time points were named T1, T2, T3. etc. Additional simultaneous data collection included: mean arterial blood pressure (MAP), HLM pump flow, noradrenaline infusion rate, arterial CO_2_ partial pressure (PaCO_2_), and body temperature.

Static cerebral autoregulation during CPB was assessed as follows: percentage changes of MAP and MCAV in relation to the corresponding preceding values were computed for each ten-minute assessment, and an autoregulatory index (AI) was calculated as pressure-flow velocity relationship (AI = MCAV [% change]/MAP [% change]), similar to what was described by Zazulia et al., who used CBF instead of MCAV [6]. An AI that approximates 0 would indicate intact cerebral autoregulation. Time points where MAP changes were < 20% or where PaCO_2_ changed by an arbitrarily chosen cutoff value of more than 20% were excluded from calculation of AI to ensure validity [7].

Prevalence of postoperative delirium was assessed in extubated patients on the ICU using the CAM-ICU test (Confusion Assessment Method for the Intensive Care Unit). A german version of the assessment that has been validated for ventilated as well as extubated patients following cardiac surgery has been used [8]. Its components are described in detail elsewhere [9]. The CAM-ICU was performed twice every day by the same investigator that was blinded for the TCD data to exclude any observational bias. According to the criteria, patients were rated as having developed POD if they were CAM-ICU positive for at least one single examination within 48 h after extubation (i.e., in the immediate postoperative period, see also Evered et al. [10]). Patients were followed up with the Richmond Agitation Sedation Scale (RASS) to assess acute onset or fluctuation of mental status changes [11]. POD was diagnosed by the occurrence of inattention in combination with either an altered level of consciousness or disorganized thinking [9].

Based on data on the known prevalence of POD in cardiac surgery patients, it was planned to include at least 45 patients into the observation [12]. All data were transferred into MS Excel (Microsoft Corp., Redmond, USA). Statistical analysis and visualization was performed using IBM SPSS 24 (Armonk, NY, USA) and GraphPad PRISM 5 (La Jolla, CA, USA). Data are presented as mean values with standard deviation (SD) and minimum and maximum value, respectively. Outliers were identified using the method propagated by Leys et al. (absolute deviation around the median), whereby a threshold of 2.5 was used as rejection criterion [13]. Significance of intergroup differences was tested using unpaired, two-tailed Student’s t-test. For categorical data, Fisher’s exact test was used. The Pearson product-moment correlation coefficient was calculated to assess the association between the patients’ age and their baseline MCAV. *P* values < 0.05 were considered statistically significant. The datasets generated and analyzed during the current study are available from the corresponding author on reasonable request.

## Results

Between July 2016 and July 2017, a total of 47 consecutive patients were recruited to participate in the study. 3 patients were excluded from subsequent data analysis because of postoperative need for extracorporeal lung or cardiac support, respectively. Prevalence of POD in the whole cohort was 27% (12 patients). Except for one individual in the no-POD group with a history of alcoholism, no patient suffered from chronic alcohol or drug abuse. No patient showed preexisting clinically relevant cognitive impairment or dementia, and prevalence of chronic arterial hypertension was equally distributed. Table 1 gives an overview of the basic patients’ and the intraoperative details. Mean patients’ age in the whole cohort was 69 (±9.8) years. Patients that developed POD were significantly older than those without delirium. Other recorded variables (gender, weight, height, body surface area, body mass index, duration of surgery as well as of aortic cross-clamping, baseline MAP, absolute MAP during CPB, MAP relative to baseline during CPB, HLM pump flow, noradrenaline infusion rate, and arterial CO_2_ partial pressure) showed no significant differences between the two subgroups. BIS values ranged from 30 to 55 in both subcohorts with no intergroup differences. Hematocrit was significantly decreased postoperatively compared to the preoperative values (30.5 (3.7) % vs. 41.2 (3.8) %, *p* < 0.0001 (paired, two-tailed Student’s t-test)), however, neither pre- nor postoperative values nor their delta differed between no-POD and POD cohort.

**Table 1:** Patient and intraoperative details

Parameter	no delirium	delirium	*p* value
Prevalence (n)	32 (73% ^a^)	12 (27% ^a^)	
Age (years)	67 (10)	75 (5)	0.008
Male gender (n)	23 (72%)	10 (83%)	0.7 ^b^
Weight (kg)	80 (14)	81 (19)	0.94
Height (cm)	174 (10)	171 (9)	0.29
Body surface area (m^2^)	1.97 (0.2)	1.95 (0.25)	0.78
Body mass index (kg/m^2^)	26.4 (3.8)	27.5 (5.7)	0.45
Chronic arterial hypertension (n)	24 (75%)	10 (83%)	0.7 ^b^
Intraoperative details			
Duration of aortic cross-clamping (min)	91.3 (37.8)	97.6 (35.6)	0.66
Duration of surgery (min)	275.0 (71.6)	288.3 (57.3)	0.57
Hematocrit preop. (%)	41.0 (3.8)	41.8 (3.8)	0.50
Hematocrit postop. (%)	30.5 (4.0)	30.7 (2.5)	0.88
Delta hematocrit (%)	10.5 (4.7)	11.2 (3.9)	0.66
BIS	41.4 (5.1)	43.5 (4.9)	0.23
MAP _bas_ (mmHg)	63 (8.4)	69 (15.1)	0.11
MAP _abs_ (mmHg)	61 (6.7)	64 (5.8)	0.19
MAP _rel_ (%)	98 (16.1)	96 (21.9)	0.75
HLM pump flow (l/min*m^2^)	2.4 (0.29)	2.3 (0.37)	0.30
Noradrenaline (μg/min*kg)	0.11 (0.09)	0.11 (0.11)	0.96
PaCO_2_ (mmHg)	36.2 (3.7)	35.4 (4.1)	0.56
Temperature (°C)	34.5 (1.1)	34 (1.0)	0.05

Values are given as mean (±SD) of the averaged individual values

MAP = mean arterial blood pressure, HLM = heart-lung machine, BIS = bispectral index

^a^ percentage of whole cohort, ^b^ Fisher’s exact test

**Fig. 1:**
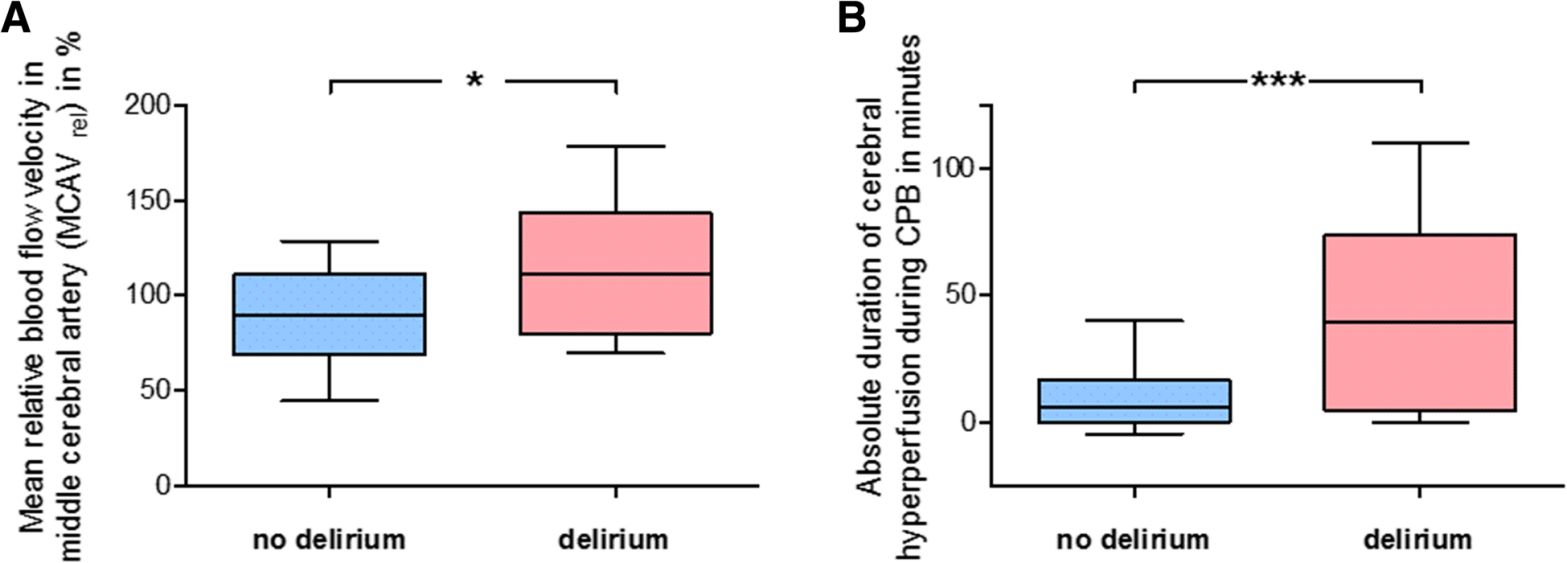
Relative blood flow velocity in middle cerebral artery during cardiopulmonary bypass. Blood flow velocity in the right middle cerebral artery (MCAV) was measured using transcranial Doppler sonography. Individual values, assessed during cardiopulmonary bypass (CPB) every 10 min, were normalized to the baseline value obtained before cannulation of the aorta and was termed MCAV_rel_. Cerebral hyperperfusion was defined as relative MCAV > 100%. **a** Mean averaged MCAV_rel_ values for the no-POD group and the POD group, respectively. **b** Duration of cerebral hyperperfusion periods during CPB in the no-POD group and the POD group, respectively.Mean ± SD (boxes) and minimum and maximum (whiskers); *n* = 32 (no delirium) and *n* = 12 (delirium); unpaired, two-tailed Student’s t-test (**b**); * *p* < 0.05, *** *p* < 0.005

Blood flow velocity in the right MCA was measured using TCD (Additional file 1: Figure S1, Supplemental Digital Content). Individual values, assessed during HLM time every 10 min, were normalized to the baseline value obtained before cannulation of the aorta and termed MCAV_rel_. Cerebral hyperperfusion was defined as MCAV_rel_ > 100%. Percentage amount of patients that experienced cerebral hyperperfusion during CPB was 32% in the subgroup without, but was 67% in the subgroup with POD, revealing a significant difference (*p* = 0.04). The mean averaged MCAV_rel_ likewise differed, as it was 90 (±21) % in the no-POD group vs. 112 (±32) % in the POD group (*p* = 0.01) (Fig. 1a). In contrast, there was no significant difference regarding the mean absolute MCAV during CPB (41.3 vs. 37.1 cm/s). Neither in the whole cohort nor in any of the subcohorts, relative changes of hematocrit correlated with the relatively changing MCAV (Pearson r for MCAV_rel_ vs. delta hematocrit: whole cohort: r = − 0.03, *p* = 0.83; no-POD: r = 0.1, *p* = 0.59; POD: r = − 0.42, *p* = 0.18).

**Fig. 2:**
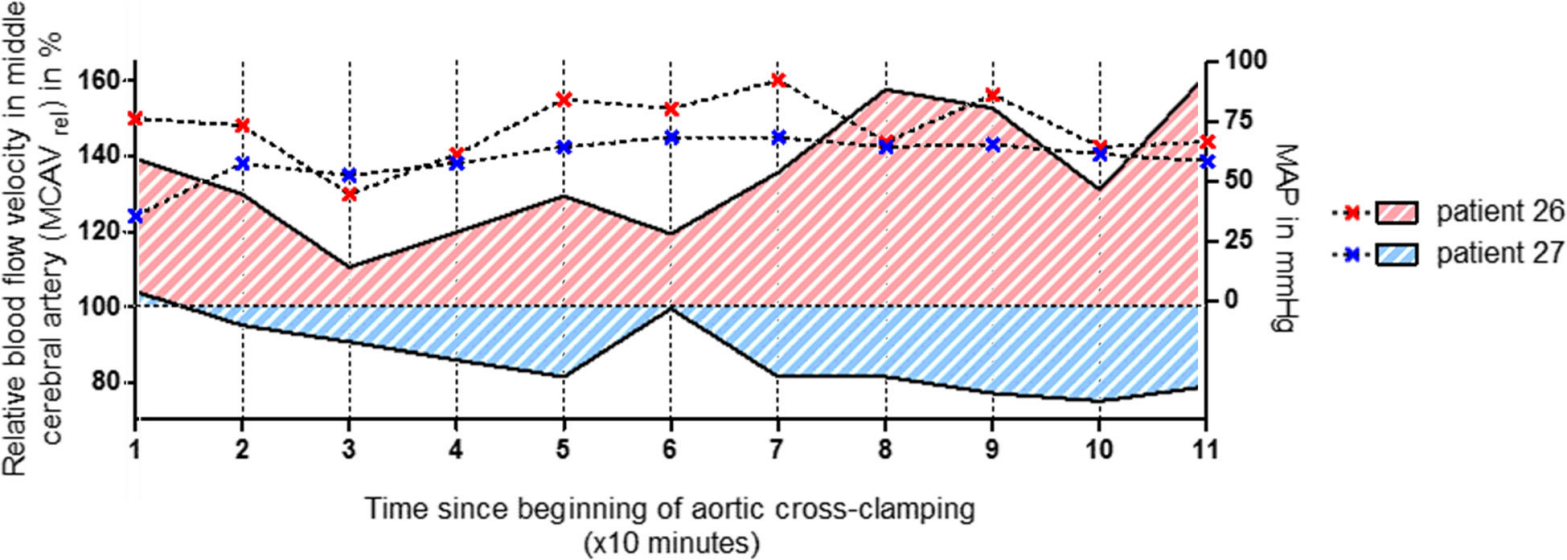
Individual relative blood flow velocities in middle cerebral artery during cardiopulmonary bypass. Blood flow velocity in the right middle cerebral artery (MCAV) was measured using transcranial Doppler sonography. Individual values, assessed during cardiopulmonary bypass (CPB) every 10 min, were normalized to the baseline value obtained before cannulation of the aorta and was termed MCAV_rel_. The figure shows the relative cerebral blood flow velocities in right MCA during CPB (solid lines, plotted on left Y axis), together with the corresponding mean arterial blood pressure (MAP) values (dashed lines, plotted on right Y axis), from two representative patients over the time. MCAV of patient no. 27, which developed no delirium, was below his individual baseline value almost the whole time during CPB. By contrast, the MCAV of patient no. 26, developing POD, was above his baseline at any time point, with peak values exceeding 160%. Note that MAP was kept within a range between 50 and 90 mmHg almost all the time in both patients

The duration of cerebral hyperperfusion was highly significantly related to the development of POD: in the subgroup that developed postoperative delirium, patients experienced cerebral hyperperfusion for about 39 (±35) min, compared to 6 (±11) min in the group without postoperative delirium (*p* < 0.001) (Fig. 1b). Normalized to the total HLM time, in the POD group, the mean percentage time of cerebral hyperperfusion was 58 (±41) %, whereas in the no-POD group, it was 9 (±22) % (*p* < 0.0001). Figure 2 visualizes the relative cerebral blood flow velocities during CPB together with the corresponding MAP values from two representative patients over the time. MAP was kept within a range between 50 and 90 mmHg almost all the time in both patients. However, the MCAV of patient no. 26, developing POD, was above his baseline at any time point, with peak values exceeding 160%. By contrast, MCAV of patient no. 27, which developed no delirium, was below his individual baseline value almost the whole time during CPB. Neither of them showed a correlation between MAP and relative MCAV (not shown).

Cerebral autoregulatory index (AI) values are given in Table 2. Overall AI in the whole cohort was 0.18, and there was no significant difference between the indices in no-POD and POD group. It has been shown that the cerebral autoregulatory capacity might be different for decreasing and increasing blood pressure [7]. In our cohort, AI calculated for decreasing MAP did not differ from that calculated for an increase in MAP (*p* = 0.97) when stable PaCO_2_ conditions have been respected. This also accounted for either subgroup.

**Table 2:** Cerebral autoregulatory index (AI)

Parameter	whole cohort	no delirium	delirium	*p* value
AI	0.18 (0.44)	0.19 (0.39)	0.17 (0.58)	0.92
AI _dcr_	0.18 (0.44)	0.16 (0.48)	0.25 (0.36)	0.63
AI _incr_	0.18 (0.59)	0.21 (0.39)	0.1 (0.9)	0.66

Values are given as mean (±SD) of the averaged individual values. P value is given for unpaired, two-tailed Student’s t-test comparing no delirium and delirium group

AI = autoregulatory index, AI _dcr/incr_ = autoregulatory index for decreasing/increasing MAP

**Fig. 3:**
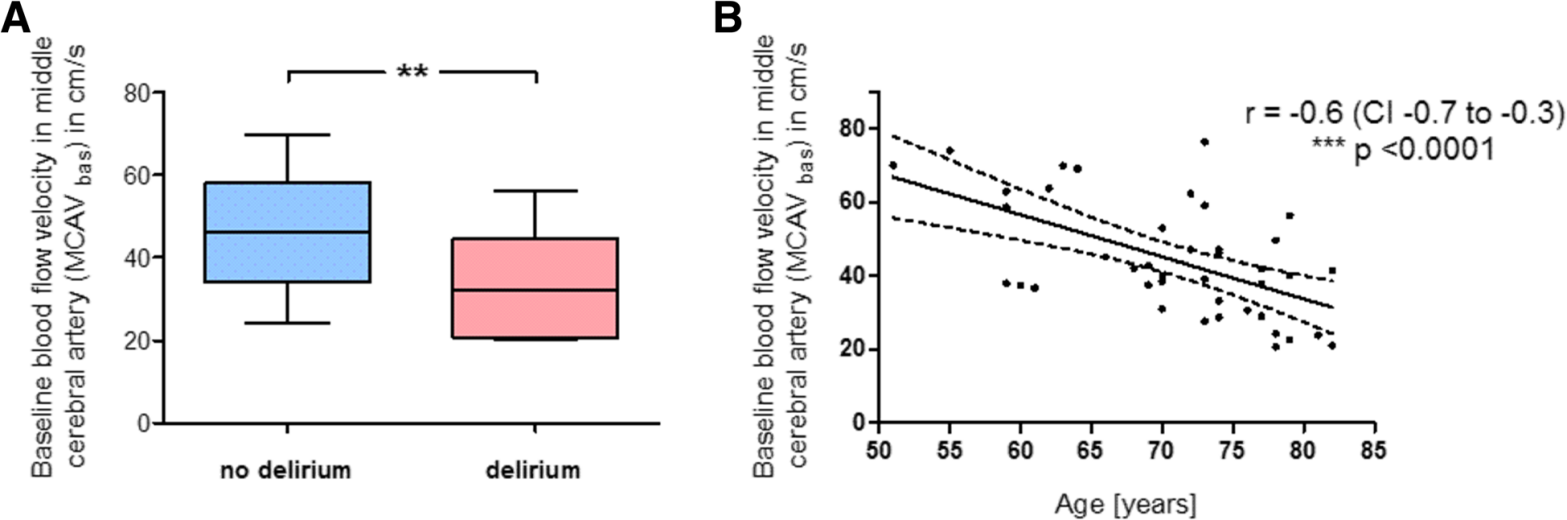
Significance of age and baseline blood flow velocity in middle cerebral artery for cerebral hyperperfusion during cardiopulmonary bypass. Blood flow velocity in the right middle cerebral artery (MCAV) was measured using transcranial Doppler sonography before cardiopulmonary bypass (CPB) and was termed MCAV_bas_. **a** Comparison of the patients’ baseline MCAV values (in cm/s), assessed before beginning of CPB, between the no-POD group and the POD group. **b** Association of the patients’ age with their measured MCAV_bas_ values, with elderly patients having a reduced baseline blood flow velocity (Pearson product-moment correlation). The dashed curved lines indicate the confidence band. r = Pearson product-moment correlation coefficient, CI = confidence interval; mean ± SD (boxes) and minimum and maximum (whiskers); n = 32 (no delirium) and n = 12 (delirium); unpaired, two-tailed Student’s t-test (**a**); ** *p* < 0.01, *** p < 0.005

Since not absolute but relative cerebral blood flow velocities during CPB differed between the two subgroups, we compared the patients’ baseline MCAV values, assessed before beginning of CPB and used as reference. Mean MCAV_bas_ was significantly lower in patients that developed postoperative delirium (32.4 (±12.0) cm/s) than in those without POD (46.1 (±11.9) cm/s; *p* = 0.002) (Fig. 3a). As there was also a difference in the patients’ age (Table 1), we found a significant correlation between age and MCAV_bas_, with elderly patients having a reduced baseline blood flow velocity (r = − 0.6, *p* < 0.0001) (Fig. 3b). We then divided the whole cohort using the median baseline MCAV, which was 40.1 cm/s, to focus only on those patients with reduced basal CBF (i.e. with MCAV_bas_ < 40.1 cm/s). This revealed 21 patients. Most of the POD patients (9 out of 12) belonged to this subcohort, but the majority (8 out of those 9 patients) was actually exposed to hyperperfusion during CPB, while only one had MVAC_rel_ values below 100%. In other words, prevalence of POD in the subcohort was 17%, when relative MCAV during CPB was kept below 100%, but it markedly increased to 53% when these patients experienced relative cerebral hyperperfusion during CPB, suggesting that not reduced basal CBF alone but too high MCAV_rel_ (i.e. cerebral hyperperfusion) promotes postoperative delirium.

## Discussion

CPB during cardiac surgery has been demonstrated to cause alterations of the cerebral blood flow [14]. The main objective of this study was to evaluate if such changes in brain perfusion, as assessed by TCD, are related to the development of postoperative delirium, being one of the most frequent neuropsychologic complications in cardiac surgery. Our analyses revealed that patients with a reduced baseline MCAV, measured before CPB, are prone to experience relative cerebral hyperperfusion during CPB, when pump flow of the HLM is adjusted according to body weight and height. These patients demonstrated a significantly higher prevalence of delirium during the early postoperative period.

In the healthy adult brain, under physiological and normal intracranial pressure conditions, CBF is maintained constant over a definite range of perfusion pressure, i.e. systemic blood pressure. This phenomenon, termed cerebral autoregulation (CAR), ensures a CBF of about 50 ml per 100 g of brain tissue per minute, given that perfusion pressure is within a range of 60 to 160 mmHg [15]. During mild hypothermia for cardiac surgery, it is accepted that autoregulation is preserved [16]. In our study, overall static AI was 0.18 for the whole cohort, with no significant differences between the two groups, suggesting intact CAR in our patients. Hypothermia was very mild, as the mean body temperature was around 34 °C in both subgroups. Furthermore, PaCO_2_ values did not differ among no-POD and POD patients.

Delirium is one cardinal complication in cardiac surgery, significantly influencing the patients’ outcome [1]. Often occurring during the early postoperative period, it accounts for prolonged lengths of ICU and hospital stay. A number of risk factors have been identified, however, the detailed underlying pathogenetic mechanisms are still elusive [2, 17]. Although it is generally assumed that neurologic or neuropsychologic complications arise from insufficient cerebral perfusion (i.e. too low CBF), there is evidence for a critical role of cerebral hyperperfusion [15]. Patel et al. demonstrated a significant decline in late postoperative cognitive function in patients whose CBF during CPB was almost doubled compared to those that were clinically unremarkable [16]. Venn et al. could confirm these findings [18]. Cerebral hyperperfusion syndrome has originally been described as a result of therapeutic interventions on arterial vessels providing blood supply to the brain, e.g., following carotid endarterectomy (CEA) [19]. Usual symptoms include a triad of headache, seizure, and transient neurologic deficits. CT or MR imaging reveals, among others, a brain edema as pathophysiologic correlate [20]. Although rare, case reports give anecdotal reference to hyperperfusion syndrome following cardiac surgery, presenting with postoperative delirium [21]. In an animal model of selective cerebral perfusion, used during aortic surgery, Haldenwang et al. could demonstrate that high CBF, compared to a low-flow regimen, resulted in cerebral edema and increasing intracranial pressure [22]. Studies on septic, non-cardiac surgery patients revealed sepsis-induced disturbed CAR, likewise being associated with delirium and suggesting a role for hyperperfusion during episodes of critical surges of arterial blood pressure that overstress the autoregulative capacity of cerebral vessels [23–26].

We performed serial studies on cardiac surgery patients that revealed a significant association of the risk for postoperative delirium with increased mean CBF velocities, as assessed by TCD during CPB, relative to the pre-bypass baseline. These increases were not limited to any specific phase of CPB. Prevalence of POD in our cohort was 27%, being in accordance with recent review literature [12]. Our findings are in line with what is described by Hori et al., who used near-infrared spectroscopy (NIRS) to determine the upper limit of CAR [4]. Their analyses revealed that excursions of the MAP above this limit were significantly associated with an increased risk for postoperative delirium. According to calculation of AI, pressure CAR was intact in our patients, and MAP values during CPB did not differ between the two groups. However, we see intergroup differences in relative cerebral blood flow. This highlights the impact of HLM pump flow rate, which may affect cerebral perfusion completely independent of other CBF-regulating parameters such as systemic blood pressure [27].

In our patients, in contrast to the relative CBF, absolute MCAV during CPB did not differ between POD and no-POD group. Therefore, basal MCAV, which was used as reference when calculating the relative hyperperfusion during CPB, was significantly lower in patients that developed POD. The patients’ age has consistently been identified as one well-documented independent risk factor for the development of POD in cardiac surgery [2, 28]. Although cerebrovascular alterations or an increased incidence of neuropsychiatric disorders in the elderly patient likely play a pathogenetic role, specific underlying mechanisms for this relation have not yet been identified. Our analyses revealed a significant association between the patients’ age and a reduced baseline MCAV, which is known from previous studies [29, 30]. We defined cerebral hyperperfusion as relative MCAV > 100%, normalized to the baseline, suggesting that age-related reduced basal MCAV is a source of hyperperfusion during CPB. However, if a low pre-bypass baseline MCAV actually increases the individual vulnerability to intraoperative hyperperfusion leading to subsequent delirium, or if it may be just a reflection of a reduced cerebral metabolism in the elderly patient that per se increases the risk for POD cannot be answered clearly by our results. We observed that the risk for POD was markedly increased particularly in those patients with reduced baseline MCAV that were actually hyperperfused during CPB, compared to those without hyperperfusion periods. Furthermore, the cumulative duration of such hyperperfusion periods was associated with the development of delirium. This all gives rise to the assumption that CPB-induced hyperperfusion may reflect one possible mechanistic link between age and POD, when HLM pump flow is adjusted only according to the patient’s body weight and height. But additional studies are urgently required, including prospective design and randomized interventional perfusion strategies (e.g., conservative CPB management vs. adjustment to continuously monitored CBF), to determine the mechanistic role of age-related reduced MCAV for the development of POD following cardiac surgery and to clarify if prevention of relative cerebral hyperperfusion during CPB may reduce the risk for POD.

The relationship between CBF and TCD-derived MCAV remains one potential methodical limitation of our findings. Since Aaaslid et al. first described the use of TCD to assess CBF in 1982, numerous authors aimed to validate this method for various clinical situations, including CPB (see also Caldas et al. [31]). During mild hypothermic CPB, results may be ambiguous, but it can be assumed that at least changes of flow velocity in the MCA reflect changes in CBF as long as arterial CO_2_ partial pressure and thus the diameter of the basal cerebral arteries remain constant [32, 33]. Therefore, assessing cerebral perfusion as well as pressure CAR during CPB using TCD appears to be valid. In addition, although the impact of usual dosages of volatile anesthetics on this relationship seems to be small, we intentionally determined baseline MCAV in anesthetized and not in awake patients prior to CPB to exclude an effect of anesthesia induction on TCD measurements [34].

Preoperative hematocrit values were significantly higher than those obtained postoperatively. Although its influence is rather small, compared to pump flow or systemic blood pressure, changing hematocrit may alter CBF [35, 36]. If hemodilution affects the validity of TCD to evaluate CBF during CPB is still uncertain. While some authors showed that during deep hypothermic CPB, CBFV increases with decreasing hematocrit [37], others have demonstrated that under conditions of laminar flow, the linear association between flow and velocity is not altered by changes in hematocrit in clinically relevant ranges [38]. Moreover, non-pulsatile flow during CPB itself may reduce the impact of hemodilution on CBFV [39]. Thus, the findings of Paut and Bissonnette “[...] support the use of transcranial Doppler sonography to estimate cerebral blood flow [...] during bypass.” [38]. Furthermore, it has been shown that cerebral autoregulation is preserved even with decreasing hematocrit as long as PaCO_2_ is held within normal ranges [40]. In our patients, pre- as well as postoperative hematocrit values in the POD group equaled those in the no-POD group. Furthermore, neither in the whole cohort nor in any of the subcohorts, relative changes of hematocrit correlated with the relatively changing MCAV. Together with the observed association of subsequent POD not only with increased relative CBFV but also its duration, this makes a relevant effect of intraoperative hemodiluation on our findings highly unlikely.

In addition, other important possible confounders such as preexisting micronutrient deficiencies or clinically inconspicuous cognitive impairment cannot be ruled out with absolute certainty, as comprehensive preoperative tests (e.g., Nutritional Risk Screening (NRS), see also Ringaitienė et al. [41]) have not been performed. Furthermore, due to the short follow-up period of 48 h following extubation, we have no data on the possible development of postoperative cognitive dysfunction (POCD), which is said to occur weeks, months or even years after surgery, in contrast to POD, which is seen during the immediate postoperative period [10]. Further studies with extended follow-up are needed.

## Conclusions

Taken together, our results suggest the necessity of a more individualized hemodynamic management during CPB, especially in the population at risk. Systemic blood flow velocity significantly affects CBF, independent of perfusion pressure. During CPB, HLM pump flow may be easily adapted, and we recommend integrating dynamic parameters such as measured patient’s individual baseline CBF instead of using body surface area when adjusting pump flow rate. TCD can be a useful intraoperative guiding instrument for adequate hemodynamic parameters.

## Additional file

Additional file 1: **Figure S1**: Assessment of blood flow velocity in middle cerebral artery by transcranial Doppler sonography **(A)** Circle of Willis and position of the ultrasound probe for insonation of the right middle cerebral artery via a temporal window. (from: Wikimedia Commons. Image courtesy of Rune Aaslid (user name: Runeaaslid). The file is licensed under the Creative Commons Attribution 3.0 Unported license. Permission is granted to copy, distribute and/or modify under the terms of the GNU Free Documentation License, Version 1.2 or any later version.) **(B)** Color Doppler (upper window) and pulsed-wave Doppler sonography (lower window) of the M1 segment of the middle cerebral artery. Measured blood flow velocity was averaged over time (time-averaged peak velocity, TAPV). (TIF 4242 kb)

## Abbreviations

AI: Autoregulatory index
BIS: Bispectral index
CAM-ICU: Confusion Assessment Method for the Intensive Care Unit
CAR: Cerebral autoregulation
CBF: Cerebral blood flow
CEA: Carotid endarterectomy
HLM: Heart-lung machine
ICU: Intensive care unit
MCA: Middle cerebral artery
MCAV: Blood flow velocity in MCA
NIRS: Near-infrared spectroscopy
PaCO_2_: Arterial CO_2_ partial pressure
POD: Postoperative delirium
TCD: Transcranial Doppler sonography
