# Supplementary figures and images for: Relative cerebral hyperperfusion during cardiopulmonary bypass is associated with risk for postoperative delirium: a cross-sectional cohort study
Source: BMC Anesthesiol. 2019 Mar 9;19:35. doi: 10.1186/s12871-019-0705-y (PMC6408763; doi:10.1186/s12871-019-0705-y)

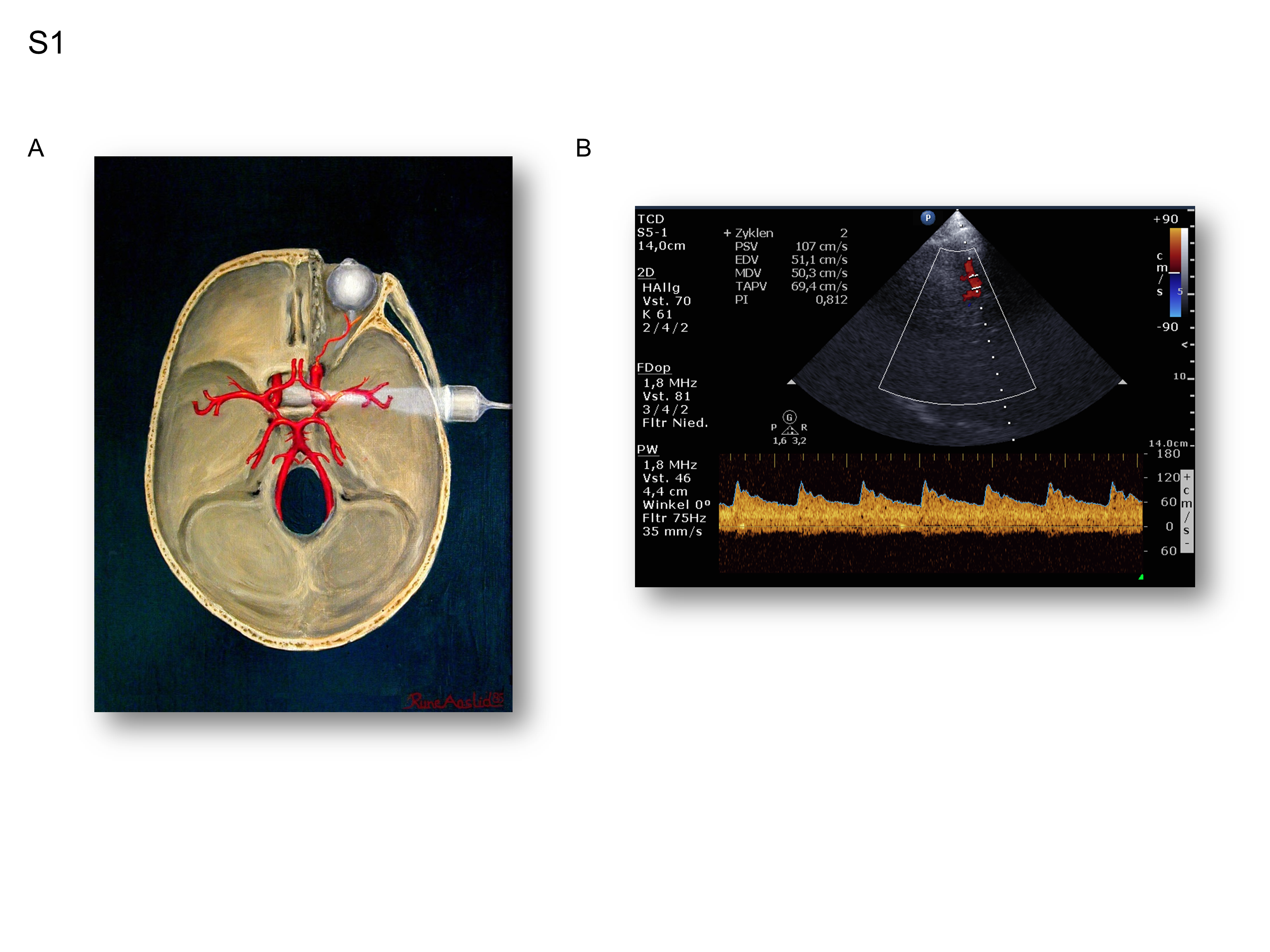

Supplement: Supplementary file 1 — Figure S1: Assessment of blood flow velocity in middle cerebral artery by transcranial Doppler sonography (A) Circle of Willis and position of the ultrasound probe for insonation of the right middle cerebral artery via a temporal window. (from: Wikimedia Commons. Image courtesy of Rune Aaslid (user name: Runeaaslid). The file is licensed under the Creative Commons Attribution 3.0 Unported license. Permission is granted to copy, distribute and/or modify under the terms of the GNU Free Documentation License, Version 1.2 or any later version.) (B) Color Doppler (upper window) and pulsed-wave Doppler sonography (lower window) of the M1 segment of the middle cerebral artery. Measured blood flow velocity was averaged over time (time-averaged peak velocity, TAPV). (TIF 4242 kb) [file 12871_2019_705_MOESM1_ESM.tif]
